# Medication-associated thyroid eye disease: a review

**DOI:** 10.1007/s00417-025-07089-w

**Published:** 2026-01-17

**Authors:** Terence Ang, Abdullah Almater, Dinesh Selva

**Affiliations:** 1 Discipline of Ophthalmology and Visual Sciences, The University of Adelaide, Adelaide, SA Australia; 2 Department of Ophthalmology, The Royal Adelaide Hospital, Adelaide, SA Australia; 3 Research Excellence Center in Ophthalmology and Visual Sciences, Department of Ophthalmology, College of Medicine, King Saud University, Riyadh, Saudi Arabia; 4 King Saud University Medical City, King Saud University, Riyadh, Saudi Arabia

**Keywords:** Thyroid eye disease, Thyroid-associated orbitopathy, Graves disease, Thyroid dysfunction, Alemtuzumab, Immune checkpoint inhibitors, Lenalidomide

## Abstract

**Purpose:**

To systematically review the literature surrounding medication-associated thyroid eye disease (TED).

**Methods:**

A systematic search was conducted from inception to the 31st of March 2025 on PubMed, EMBASE and Web of Science. Studies describing medication-associated thyroid dysfunction and the subsequent development of TED were included. Articles without thyroid dysfunction and/or the absence of orbitopathy were excluded.

**Results:**

A total of 23 studies met the inclusion criteria. Implicated medications included alemtuzumab, immune checkpoint inhibitors (ICI), amiodarone and lenalidomide. The mean onset of thyroid dysfunction from commencement of alemtuzumab was 26.3 ± 12.2 months (Range: 7 to 53.3 months); and the mean onset of developing orbitopathy from thyroid dysfunction was 19.9 ± 21.4 months (Range: 0 to 96 months). Graves’ disease was the most common condition resulting in the development of alemtuzumab-associated TED. ICIs reported to cause TED included ipilimumab (anti-CTLA-4 drug), tremelimumab (anti-CTLA-4 drug), durvalumab (PD-1 inhibitor) and nivolumab (PD-1 inhibitor). Anti-CTLA4 drugs, such as ipilimumab and tremelimumab, were the most common agents implicated in the development of ICI-associated TED. ICIs may also incite orbital inflammation in the absence of thyroid dysfunction. A single case-report of amiodarone- and lenalidomide-associated TED was noted.

**Conclusion:**

Medication-associated TED is an important differential consideration in the work-up of orbital inflammatory disease. The clinico-radiological manifestations and the principles of management of medication-associated TED were similar to that of *de novo* TED. Certain medications, such as various ICIs, may demonstrate different clinical phenotypes of orbital inflammation, inciting either TED or a “TED-like” orbitopathy without evidence of underlying thyroid dysfunction.

## Key messages

**What is known?**

- Orbital inflammatory disease (OID) has a wide range of aetiologies and may be secondary to various medication adverse effects.

**What is new?**

Medication-associated thyroid eye disease (TED) represents an important pattern of involvement within the spectrum of OID.

- Medications which may cause TED include: Alemtuzumab (anti-CD52 monoclonal antibody), various immune checkpoint inhibitors (ICI), amiodarone and lenalidomide.
- Medication-associated TED is an important differential consideration in the work-up of OID and is relevant to clinicians across a range of disciplines.

## Introduction

Thyroid eye disease (TED) is the most common cause of orbital disease. TED most commonly arises due to Graves’ disease, however, may occur in hypothyroidism and euthyroid states. These thyroid disorders are typically *de novo* autoimmune processes, however, rarely, they may arise secondarily as medication adverse effect (AE). Medication-associated TED is an important diagnostic consideration in orbital inflammatory disease but also sheds light on the various pathogenic mechanisms underlying TED. Medication-associated TED is rare and has been described as an AE for several medications, particularly the class of monoclonal antibodies. Herein this systematic review, we identify the range of medications which may cause TED, and summarize their specific clinico-radiological features, management and clinical outcomes.

## Methods

This systematic review was conducted in accordance with the Preferred Reporting Items for Systematic Reviews and Meta-Analyses (PRISMA) guidelines. A comprehensive systematic search was conducted from inception to the 31^st of^ March 2025 on PubMed, EMBASE and Web of Science databases (Appendix Table 3). Inclusion criteria included patients of all ages with medication-associated TED, defined as medication-associated thyroid dysfunction and subsequent development of TED. Thyroid dysfunction was evidenced by abnormal thyroid function tests (including thyroid autoantibodies such as TR-Ab, TPO-Ab and/or Tg-Ab), whilst TED was evidenced by the range of characteristic orbital clinical and/or radiological features (e.g.: proptosis, bilateral extraocular muscle enlargement, lacrimal gland enlargement, etc.) [1–3]. There was exclusion of patients without evidence of thyroid dysfunction (i.e. deranged thyroid function tests and/or elevated thyroid autoantibodies) and/or absence of orbitopathy, patients with pre-existing thyroid dysfunction or orbitopathy prior to commencement of an inciting medication. Abstract, posters and conference articles were also excluded. Articles were screened by the authors (TA and AA), followed by a full-text screen (TA and AA). Patient-level data, where available, was extracted including patient demographics, clinical features, time of onset between administration of medication to thyroid dysfunction, time of onset between thyroid dysfunction to TED, management and clinical outcomes. Where applicable, results are expressed as means ± standard deviation (σ) and presented in relevant tables.

## Results

**Fig. 1:**
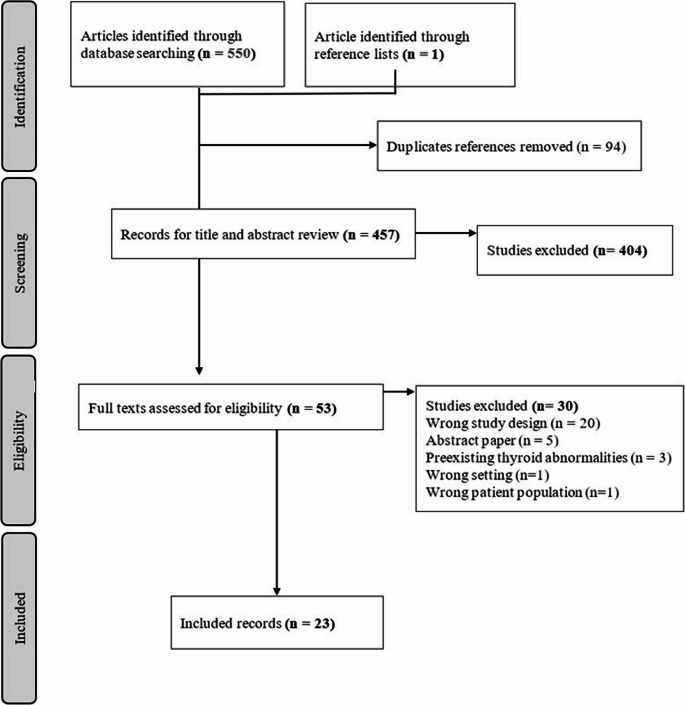
Preferred reporting items for systematic reviews and meta-analyses (PRISMA) flowchart for inclusion and exclusion of study articles

A total of 457 studies were screened from PubMed, EMBASE and Web of Science, yielding 23 studies meeting the inclusion criteria (Fig. 1). Medications identified within the literature to cause TED include: Alemtuzumab (anti-CD52 monoclonal antibody), various immune checkpoint inhibitors (ICI), amiodarone and Lenalidomide. ICIs reported to cause TED include ipilimumab (anti-CTLA-4 drug), tremelimumab (anti-CTLA-4 drug), durvalumab (PD-1 inhibitor) and nivolumab (PD-1 inhibitor).

### Alemtuzumab

Alemtuzumab is a monoclonal antibody targeting CD52, which is primarily used in the treatment of multiple sclerosis (MS). When indicated for MS, alemtuzumab is administered intravenously, typically with the first course over 5 consecutive days (12 mg/Kg) followed by a 3-day course (12 mg/Kg) 1-year later. The pathogenesis and clinico-radiological features of alemtuzumab-associated TED remain poorly defined, secondary to its rare incidence. Autoimmune thyroid disease (ATD), also referred to as autoimmune thyroid events, have been reported to occur with alemtuzumab. It is a dose and frequency independent risk, and almost exclusively occurs when alemtuzumab is used in MS patients [4, 5]. The pathogenesis of alemtuzumab-associated thyroid disease is hypothesized to be related to the immune reconstitution period following alemtuzumab, whereby there is rapid repopulation of immature B lymphocytes and homeostatic T-lymphocyte proliferation. These are the same mechanisms underlying the efficacy of alemtuzumab in reducing MS disease progression [6]. Prior studies have attempted to define the incidence of TED as an AE, with prior reports ranging between 10.2% to 16% [7, 8]. Scappaticcio et al. presented a large (*n* = 1362) systematic review and meta-analysis with pooled data of MS patients (without prior thyroid dysfunction) treated with alemtuzumab. ATD was noted in 33% of patients treated with alemtuzumab, of which the majority were Graves disease (GD) (63%). There was a pooled prevalence of 11% of orbitopathy in GD patients [4].

A total of 14 studies, including 56 patients, reported alemtuzumab-associated TED, of which the earliest report dated to 2013 by Aranha et al. (Table 1) [6–20]. 8 studies (*n* = 23 patients) presented patient-level data for data extraction and analysis [7, 9, 10, 12–15, 20]. All received alemtuzumab for management of MS, except for one patient who had alemtuzumab as part of pre-bone marrow transplant conditioning for Fanconi anemia (FA). Excluding the case of FA, the mean age at time of presentation with orbitopathy was 39.5 ± 6.8-years-old (Range: 28 to 52-years-old), and there was a female predominance (Females: 13). This patient demographic is consistent with the predisposition of MS to affected young-middle aged females. Where reported, the mean onset of thyroid dysfunction from commencement of alemtuzumab was 26.3 ± 12.2 months (Range: 7 to 53.3 months); and the mean onset of thyroid dysfunction to development of orbitopathy was 19.9 ± 21.4 months (Range: 0 to 96 months). Ueland et al. reported pooled data of nine patients with alemtuzumab-associated TED from a total of fifty-eight patients with GD. Within this study, GD was observed at a median of 40.5 months after alemtuzumab, and TED was observed at a median of 17 months after GD diagnosis [16].

**Table 1:** Summary table of alemtuzumab-associated thyroid eye disease

Author (Year published)	Patient-level data available?	Sample (patients)	M: F	Mean age	Indication for ALZ	Mean onset of GD from ALZ	Mean onset of ALZ-TED from GD (months)	Management
Aranha et al. (2013)	Yes	1	0:1	36	MS	27	9	Thyroidectomy and conservative ocular lubricants
Daniels et al. (2014)	Pooled study	4	NS	NS	MS	NS	NS	NS
Tsouridi et al. (2015)	Yes	2	1:1	34.5	MS	30.5	NS	Thyroidectomy (*n* = 2) Thiamazole and propranolol (*n* = 2) Methylprednisolone (*n* = 1)
Trinh et al. (2016)	Yes	1	0:1	36	MS	24	12	Selenium (*n* = 1)
Pariani et al. (2018)	Pooled study	7	NS	NS	MS	NS	NS	NS
Roos et al. (2019)	Yes, for 6 out of 10 cases	10	4:2	42.7	MS	14.8	34.5	Corticosteroids (*n* = 3) Orbital decompression (*n* = 1) Thyroidectomy (*n* = 1) Other immunosuppression (*n* = 1)
Sovetkina et al. (2020)	Pooled study	2	NS	NS	MS	NS	NS	NS
Kazakou et al. (2021)	Pooled study	4	NS	NS	MS	NS	NS	No treatment for all patients
Manso et al. (2022)	Pooled study	2	NS	NS	MS	NS	NS	Corticosteroids and rituximab (*n* = 1)
Nirmalan et al. (2023)	Yes	5	2:3	43.8	MS	32.2	15.8	Selenium (*n* = 2) Orbital decompression (*n* = 2) Thyroidectomy (*n* = 3) Teprotumumab (*n* = 1) Tocilizumab (*n* = 1)
Rodriguez et al. (2024)	Yes	6	3:3	36	MS	30.5	15.2	Selenium (*n* = 2) Corticosteroids (*n* = 1) Thyroidectomy (*n* = 3) Tocilizumab (*n* = 2) Sarilumab (*n* = 1)
Muller et al. (2024)	Yes	1	0:1	37	MS	24	12	Rituximab (*n* = 1) Corticosteroids (*n* = 1) Thyroidectomy (*n* = 1)
Ueland et al. (2024)	Pooled study	9	NS	NS	MS	NS	Median: 17	Corticosteroids (*n* = 2)
Cima et al. (2018)	Yes	1	1:0	13	Conditioning for BMT for FA	36	7	Selenium (*n* = 1) Corticosteroids (*n* = 1)

Key: *M* male, *F* female, *ALZ* alemtuzumab, *TED* thyroid eye disease, *NS* not specified, *MS* multiple sclerosis, *BMT* bone marrow transplant, *FA* Fanconi anemia

Alemtuzumab-associated TED was bilateral in all cases but may demonstrate some asymmetrical orbital involvement. Similar to *de novo* TED, clinical hallmarks of alemtuzumab-associated TED included symptoms such as conjunctival injection, orbital pain, periorbital oedema and/or erythema, diplopia, and painful ocular motility, and signs such as chemosis, proptosis, lid retraction and restricted ocular motility. Clinical severity ranged from mild to severe inflammation, as rated by the clinical activity score (CAS) or vision, inflammation, strabismus, and appearance score (VISA). Where reported, the mean presenting CAS score was 3.6 (Range: 1 to 8). Hyperthyroidism with elevated thyroid autoantibodies, consistent with GD, was the most common pattern of thyroid dysfunction, whilst euthyroid, and hypothyroid states were less common.

The radiological appearance of alemtuzumab-associated TED is similar to that of *de novo* TED, in which there may be proptosis, bilateral diffuse EOM enlargement with tendon sparing and potentially associated optic nerve compression. Involvement of the lacrimal gland was not reported in any study. However, specific radiological characterization within reports remains limited and vastly undetailed compared to those of *de novo* TED.

The management of alemtuzumab-associated TED is focused on achieving a euthyroid state, along with tailoring ophthalmopathy-specific treatment according to clinical severity. Conservative medical management was pursued in the majority of cases and ranged from conservative ocular lubrication and selenium supplementation in mild cases, to pulsed intravenous (IV) methylprednisolone as per the European Group on Graves’ orbitopathy (EUGOGO) protocol or oral prednisolone, for more severe inflammation. In some refractory cases, there was escalation of immunosuppression with agents such as tocilizumab, teprotumumab, rituximab and other biological agents (e.g.: cyclosporine, tacrolimus, mycophenolate). Surgical intervention via orbital decompression was conducted in four patients [7, 8, 20]. Thyroidectomy/radioactive iodine ablation was performed in twenty-one patients to achieve an euthyroid state. Long-term clinical outcomes were poorly reported within studies. Additionally, there remains limited comparative data between the risk factors, clinical features, and outcomes between medication-associated TED and *de novo* TED.

### Immune checkpoint inhibitors

Immune checkpoint inhibitors (ICIs), such as those targeting PD-1, PD-L1 and CTLA-4, are a form of immunotherapy used in the treatment of various malignancies and are associated with a range of immune-related adverse events (irAE). These include ophthalmic AE such as intraocular inflammation (i.e.: uveitis) and rarely, orbital inflammation. Other irAEs include thyroid dysfunction, hypophysitis, myocarditis, pneumonitis and colitis. The clinico-radiological characteristics of ICI-associated ophthalmopathy have been reported in prior reviews [19]. Notable examples of ICI-associated orbital inflammation include Ipilimumab (anti CTLA-4), Tremelimumab (anti CTLA-4), nivolumab (anti-PD1) and Durvalumab (anti-PD1). However, a key consideration to highlight is the delineation between ICI-associated TED and orbital inflammation occurring without any underlying thyroid dysfunction. There are vast reports of ICI-associated orbital inflammation, however, many may report normal thyroid function or do not specify serum thyroid function or autoantibodies [21–37].

Within the focus of this systematic review, we draw attention to ICI-associated TED, whereby there is evidence of underlying thyroid dysfunction. This review notes seven studies, with patient-level data for seven patients, which reveal orbitopathy in the presence of thyroid dysfunction as a result of an ICI irAE (Table 2). Patients were typically older (Mean age: 57 ± 17.9-years-old) and 57.1% were male. The most common indication for commencement of ICI was metastatic melanoma. Ipilimumab (*n* = 3) and Tremelimumab (*n* = 3), both anti-CTLA-4 drugs, were the most commonly implicated ICI [38–44]. Durvalumab was reported in one case, but in combination therapy with Tremelimumab [42].

**Table 2:** Summary table of immune-checkpoint inhibitor-associated thyroid eye disease

Author (Year published)	Patient-level data available?	Sample (patients)	M: F	Mean age	Indication for ICI	ICI	Mean time of onset from commencement of ICI to TED (months)	Management
Borodic et al. (2011)	Yes	1	1:0	51	Melanoma	Ipilimumab	NS; second dose	Corticosteroids (*n* = 1) Cantholysis (*n* = 1)
Bronstein et al. (2011)	Yes, for 1 patient	1	1:0	51	Melanoma	Tremelimumab	8	Corticosteroids (*n* = 1)
Min et al. (2011)	Yes	1	0:1	51	Melanoma	Ipilimumab	NS; four doses	Corticosteroids (*n* = 1)
Ricciuti et al. (2017)	Yes	1	0:1	63	Non-small cell lung cancer	Nivolumab	14	Corticosteroids (*n* = 1)
Sabini et al. (2018)	Yes	1	1:0	70	Lung adenocarcinoma	Tremelimumab and Durvalumab	1	Corticosteroids (*n* = 1)
Sagiv et al. (2019)	Yes	1	1:0	51	Melanoma	Tremelimumab	6	Corticosteroids (*n* = 1)
Sun et al. (2021)	Pooled data from 7 patients with orbital complications; 1 patient with TED	1	0:1	62	Melanoma	Ipilimumab	NS	Corticosteroids (*n* = 1)

Key: *M* male, *F* female, *ICI* immune checkpoint inhibitor, *TED* thyroid eye disease, *NS* not specified

Symptom onset varies, ranging from 1 to 14 months, after commencement of ICI and following repeated infusions. The clinico-radiological appearance of ICI-associated TED is typically bilateral diffuse EOM enlargement with anterior tendon sparing, similar to *de novo* TED. However, herein lies a diagnostic subtlety, in that ICIs may induce a “TED-like” orbitopathy without evidence of any underlying thyroid dysfunction [45–47]. The exact pathogenesis of ICI-associated orbital inflammation is not fully understood, however, may be related to the underlying immunogenic pathways influenced by the ICIs, resulting in T-cell mediated inflammation. Due to the underlying role of genetic factors in the pathogenesis of *de novo* TED, prior literature has also investigated the role of the CTLA-4 gene polymorphisms and its associations with autoimmune thyroid disease, including Graves’ disease and Hashimoto’s thyroiditis [48, 49]. CTLA-4 is a negative regulator of T-cell activation, and the proposed hypothesis is that a reduction in CTLA-4 expression or function, attenuated by the anti-CTLA-4 drugs, can cause T-cell proliferation and development of autoimmune disease [50, 51]. A meta-analysis by Wang et al. revealed a significant association between a specific polymorphism (+ 49 A/G) on the CTLA-4 gene and the development of TED [50]. ICIs, such as ipilimumab (anti-CTLA-4), have been observed to cause an orbital inflammation in the absence of thyroid dysfunction, yet may also lead to thyroid dysfunction and the subsequent development of TED as a potential irAE. There may be certain delineating features between these underlying entities, as ICI-associated orbital inflammation without underlying thyroid dysfunction may occasionally present as an inflammatory mass/lesion involving the EOM, tendon involvement and unilateral orbital myositis- features which are not typical or consistent with TED [21, 52]. Thus, thyroid function tests play an integral role in delineating the exact clinico-radiological phenotype of ophthalmopathy which may arise as an AE of ICI therapy. To further add to the diagnostic complexity, in some situations, systemic signs and serological evidence of thyroid dysfunction may only develop years following an initial presentation with ICI-associated TED (initially, positive anti-microsomal and anti-thyroglobulin antibodies and TSH-R antibody, but euthyroid). Thus, clinicians should consider baseline thyroid function tests prior to commencing ICIs; autoimmune thyroid markers in the initial orbital assessment; and follow-up clinical assessment and thyroid function tests in patients with ophthalmopathy [38, 53].

ICI-associated TED has been managed with systemic corticosteroids with response and success, however, there is limited long-term follow-up, primarily due to the rates of mortality related to the patients’ underlying malignancy. There remains limited information regarding the role of achieving euthyroid states via medical or surgical intervention in ICI-associated TED.

### Amiodarone

Amiodarone is an antiarrhythmic cardiac medication that may cause thyroid dysfunction, including hyper- and hypo-thyroidism. Orbitopathy is not a typical feature of thyrotoxicosis due to amiodarone therapy, and there have been sparse cases and literature surrounding the development of TED due to amiodarone. Rabinowe et al., Wilson et al. and Sundelin et al. each report a case of amiodarone-associated orbitopathy, however, only the case described by Rabinowe et al. met inclusion criteria as defined in this current review [54–56]. In each case, there were features of bilateral periorbital oedema, lid-lag, and orbital signs, such as proptosis, restricted ocular motility. Rabinowe et al. describes a case of bilateral extraocular muscle enlargement following the commencement of amiodarone, with positive anti-microsomal antibodies (i.e. Anti-thyroid peroxidase [TPO] antibodies). In the case described by Sundelin et al. there was euthyroid disease with extraocular muscle enlargement, but no evidence of thyroid dysfunction or thyroid autoantibodies, whilst in Wilson et al.’s case orbital imaging was not conducted despite abnormal thyroid function and elevated TR-Ab. Management included reducing the amiodarone dose and achieving an euthyroid state. Additionally, Wilson et al. reported good response and complete resolution following eight cycles of Teprotumumab.

### Lenalidomide

Lenalidomide is an immunomodulatory imide drug (Cereblon modulator), primarily used in the treatment of multiple myeloma. To date, there has been a single case-report of lenalidomide-associated orbital inflammation with associated thyroid dysfunction. Slean et al. reports a 76-year-old female who developed right upper lid retraction, proptosis, and elevated thyroid autoantibodies including thyrotropin receptor antibody, thyroglobulin antibody and thyroid peroxidase antibody, following 21 doses of lenalidomide [57]. The patient was otherwise serologically euthyroid. MRI revealed right EOM contrast-enhancement. Monitoring over 10-months revealed persistently elevated thyroid autoimmune markers and persistent right proptosis and upper lid retraction. Within this isolated report, it is difficult to assess the exact association between the evident thyroid dysfunction and the development of orbital symptoms. Despite the elevated thyroid autoantibodies, the patient was euthyroid with a unilateral orbital process. Lenalidomide is well associated with autoimmune thyroid disease, particularly hypothyroidism. With the growing evidence of medication-associated euthyroid Graves’ disease, it remains highly plausible that the orbital features may be related to sub-clinical hyperthyroidism caused by lenalidomide. Other potential hypotheses of thyroid dysfunction noted by the authors include the patient’s underlying multiple myeloma [57].

## Discussion

It is important to note the distinction between medication-associated orbital inflammation and medication-associated TED, whereby there is evidence of underlying thyroid dysfunction caused by the inciting medication. In the diagnostic approach of orbital inflammatory disease, even when suspecting an inciting medication, delineating an underlying thyroid disorder such as those underlying the aforementioned medications is important. This key feature helps guide further systemic management such as thyroidectomy and other medical therapy to achieve a euthyroid state. Thus, patients who are about to commence alemtuzumab or ICIs should have baseline thyroid function tests. The principles of management of medication-associated TED mirror those of *de novo* TED unrelated to medications. In mild disease, ocular lubricants and supplemental selenium have been utilized in alemtuzumab-associated TED. In more severe disease, systemic corticosteroids and other immunosuppressive agents remain a major part of therapy. Orbital decompression is indicated in situations of significant optic nerve compromise. The other facet of management to be considered includes achieving a euthyroid state via pharmacological (e.g.: methimazole) or surgical intervention (i.e. thyroidectomy), both of which have been reported in alemtuzumab-associated TED.

There is some overlap between medications inciting “TED-like” orbital inflammation without thyroid dysfunction, such as those observed in ICIs. Evidently, there are two potential clinico-radiological phenotypes of ICI-orbital inflammation, in that thyroid dysfunction may be present or absent. In those cases with underlying thyroid dysfunction, the clinico-radiological features mimic those of *de novo* TED. Meanwhile, in the absence of thyroid dysfunction, patients may demonstrate a “TED-like” orbital presentation, but may also present with unilateral, tendon involvement and an orbital mass/lesion associated with the EOMs- features atypical of TED. Many prior reports of ICI-associated ophthalmopathy may not specify thyroid function tests, and thus, this may be an under-represented entity. Furthermore, in many articles, baseline thyroid function tests and antibody status were not clearly reported, and thus, limits conclusions regarding causality. Articles were primarily limited to retrospective case-reports and small case-series and thus there is limited long-term follow-up data. Further longitudinal studies with larger cohorts are necessary to define incidence, risk factors, and outcomes of medication-associated TED. Comparative studies may also highlight the features, clinical course and outcomes of medication-associated TED compared to *de novo* TED. It is likely that the underlying immunomodulatory drug’s mechanism of action incites an immunogenic response leading to thyroid dysfunction and subsequent development of ophthalmopathy. Prior studies have explored the role of genetics, such as those investigating the CTLA-4 gene and by extrapolation, the hypothesized mechanisms in which anti-CTLA-4 drugs may lead to increased irAE. Further studies are necessary to understand the various genetic and immunomodulatory pathways at play in the development of medication-associated TED. In doing so, we expand our understanding of the various pathways underlying orbital inflammation in *de novo* TED.

Furthermore, this review noted literature surrounding lithium-associated orbitopathy. Lithium is used in the management of manic-depressive conditions, and there is established literature surrounding lithium-associated orbitopathy, presenting with proptosis and extraocular muscle enlargement. Additionally, thyrotoxicosis has been well cited as an adverse effect of lithium, and there are cases of lithium-associated orbitopathy occurring with concurrent thyroid dysfunction, such as Graves’ disease [58, 59]. However, there remains limited evidence of causation or association of lithium-associated orbitopathy with underlying thyroid dysfunction from these reports. Many historical cases have limited reports of thyroid function and thyroid autoantibodies, along with radiological reports, and thus, have not met the inclusion criteria defined in this review [58–61]. Clinicians need to remain vigilant in monitoring and identifying underlying thyroid dysfunction in the occurrence of orbital features, and orbital imaging plays an important role in diagnosis and management.

In conclusion, this systematic review provides a comprehensive summary of various medication-associated TED. Thyroid dysfunction and the subsequent development of TED is of significant clinical importance, to ensure clinicians can provide appropriate patient education, monitoring and identification of this AE, and initiate targeted management to prevent visual morbidity and compromise. Further longitudinal and large-sample cohort studies are necessary to define the incidence, risk factors and outcomes of medication-associated TED.

## Data Availability

The data that support the findings of this study are available from the corresponding author, [TA], upon reasonable request.

## Appendix

**Table 3:** Logic grid for systematic search

Thyroid Eye Disease	AND	Medication induced
“graves ophthalmopathy” [mh: noexp] OR Thyroid eye disease [tiab] OR thyroid ophthalmopathy [tiab] OR thyroid orbitopathy [tiab]		“alemtuzumab” [mh: noexp] OR alemtuzumab [tiab] OR drug induced [tiab] OR medication induced [tiab] OR “Antibodies, Monoclonal, Humanized” [mh: noexp] OR Tremelimumab [tiab] OR Durvalumab [tiab] OR “Immune Checkpoint Inhibitors” [mh: noexp] OR “Ipilimumab” [mh: noexp] OR “Lenalidomide” [mh: noexp] OR “nivolumab” [mh: noexp] OR cemiplimab [tiab] OR “pembrolizumab” [tiab] OR “atezolizumab” [tiab] OR “avelumab” [tiab] OR “ibrutinib” [tiab] OR “Interferon beta-1b” [mh: noexp] OR “growth hormone” [mh: noexp] OR “Amiodarone” [mh: noexp] OR “Lithium” [mh: noexp]

## Declarations

### Copyright and permissions

The authors do not have any copyright and permissions to declare.
